# Potential for the Bio-Detoxification of the Mycotoxins Enniatin B and Deoxynivalenol by Lactic Acid Bacteria and *Bacillus* spp.

**DOI:** 10.3390/microorganisms12091892

**Published:** 2024-09-13

**Authors:** Sandra Mischler, Amandine André, Irene Chetschik, Susanne Miescher Schwenninger

**Affiliations:** Institute of Food and Beverage Innovation, ZHAW Zurich University of Applied Sciences, 8820 Wädenswil, Switzerland; sandra.mischler@zhaw.ch (S.M.); amandine.andre@zhaw.ch (A.A.); irene.chetschik@zhaw.ch (I.C.)

**Keywords:** mycotoxins, enniatin B, deoxynivalenol, lactic acid bacteria, *Bacillus*, Fusarium, bio-detoxification, HPLC-MS/MS

## Abstract

Mycotoxins, toxic compounds produced by fungi, pose significant risks to food safety and human health. This study investigates the bio-detoxification potential of 238 strains of lactic acid bacteria (LAB) and *Bacillus* spp., previously isolated from cereals (including mycotoxin-contaminated grains), against the emerging mycotoxin, enniatin B (ENB), and the prevalent mycotoxin, deoxynivalenol (DON). Out of the tested strains, 26 demonstrated notable mycotoxin reduction capabilities, including 2 *Bacillus pumilus* and 24 *Bacillus licheniformis* strains. *B. licheniformis* strains MA572, MA695, MA696, TR174a, TR284, TR363, and TR466a degraded ENB to levels below the detection limit, and six strains reduced DON by 30–35%; *B. licheniformis* TR251b and TR374 showed the highest DON reduction with 35.7%. The most promising strains for bio-detoxification were *B. licheniformis* TR284, which achieved a 100% reduction in ENB and a 28.6% reduction in DON and *B. licheniformis* TR388 with a 97.5% reduction in ENB and a 31.9% reduction in DON. None of the tested LAB strains significantly reduced either mycotoxin. These findings highlight the promising potential of *B. licheniformis* strains in bio-detoxifying mycotoxin-contaminated cereal products. Further research into the underlying detoxification mechanisms and safety aspects is essential to develop effective bio-detoxification strategies for enhancing food safety.

## 1. Introduction

The most common natural contaminants in food and feed are mycotoxins from *Aspergillus* spp., *Penicillium* spp., or *Fusarium* spp. molds. They often produce aflatoxins as secondary metabolites, namely ochratoxin A in the case of *Aspergillus* spp. and *Penicillium* spp. and trichothecenes, including deoxynivalenol (DON), zearalenone, and fumonisin, in the case of *Fusarium* spp. [1]. These toxins are well studied and considered in food legislation. However, less so are the emerging mycotoxins, enniatin and beauvericin produced by *Fusarium* spp., whose incidence is rapidly increasing [2].

Enniatins are cyclic hexadepsipeptides containing three N-methyl amino acids and hydroxy acid groups [3]. The toxins are mainly produced by *F. acuminatum*, *F. avenaceum*, *F. oxysporum*, *F. proliferatum*, *F. sambucinum*, *F. sporotrichioides*, *F. subglutinans*, and *F. tricinctum* [4]. There are 27 different types of enniatins, the most common being enniatin A, enniatin A1, enniatin B (ENB), and enniatin B1 [4,5], with ENB considered the most prevalent emerging mycotoxin [6]. The systematic review of Kolawole et al. [2] revealed enniatin levels of 0.25–10,000 µg/kg in samples of crops, animal feed, and silage, whereas the mean level was at 7854 µg/kg. The detection frequency for enniatin was evaluated at 40%. Several studies analyzing the content of enniatins in wheat, barley, and oat crops, among others, found that the mycotoxin was present in a frequency of 20 to 100% in amounts up to 3077 µg/kg [7,8,9,10]. Despite their high prevalence in cereals and their classification as emerging mycotoxins, the levels of enniatins found in cereals and cereal-based products are not yet considered in food legislation. The European Food Safety Authority (EFSA) stated in 2014 that an acute exposure to enniatins is not of concern for human health due to a lack of data [7]. Nevertheless, scientific evidence has since proven the toxic effects of ENB as a result of chronic exposure both in humans and animals [6,8,9,10,11,12].

DON belongs to the trichothecenes and is a toxin produced by species of *F. acuminatum*, *F. equiseti*, *F. poae*, *F. sporotrichioides*, *F. graminearum*, *and F. culmorum* [13,14,15,16,17]. Levels of up to 5500 µg/kg of DON in 80–100% of crops have been determined [18]. In 2024, the European Union (EU) introduced a new commission regulation, Commission Regulation (EC) No. 2024/1022, stating that DON must be surveyed and should not exceed 750 μg/kg in cereal and processed food for direct human consumption and 150 μg/kg in baby food [19]. Commonly named vomitoxin, DON can cause acute temporary nausea, vomiting, and diarrhea accompanied with headache, dizziness, and fever [20].

Toxin-contaminated food and feed has resulted in enormous food waste and poses a high risk to human consumption. Various detoxification strategies are known for mycotoxins, such as physical methods like boiling [21], adsorption by (e.g., activated carbon) [22], irradiation [23], cold plasma treatment [24], or high-pressure treatment [25]; chemical strategies like acid or alkali treatment [26]; and oxidation and reduction [27]. However, these physical and chemical methods can lead to food quality decline and nutrient loss; changes in food composition impacting, for example, flavor; or processing properties of the treated raw materials [28]. Because few chemical and physical decontamination strategies are meeting the requirements of practical application (efficiency, safety, and cost-effectiveness, as reviewed by Liu et al. [28]), biological degradation strategies using microorganisms or enzymes are a promising alternative and offer efficiency, specificity, and environmental safety [29,30].

The enzymatic degradation of mycotoxins on one hand has been well studied for many years. Isolated from microorganisms or recombined enzymes have been proven to efficiently degrade zearalenone, deoxynivalenol, and enniatin B1, among others [31]. However, the interaction of the enzymes with other mycotoxins can affect the efficiency of the biotransformation, emphasizing the importance of interaction studies in the case of natural toxins, as demonstrated by Ivanova et al. [32].

On the other hand, microorganisms are known to either bind or degrade mycotoxins [29,33,34,35], although binding could be problematic in the event of release in the gastrointestinal tract after ingestion [36,37]. In a previous study by Mischler et al. [38], the ability of LAB and *Bacillus* spp., including strains isolated from mycotoxin-contaminated wheat grains, to reduce zearalenone was successfully demonstrated, whereas the reduction in DON was evaluated as insufficient (<15% reduction). The bio-detoxification of DON by lactic acid bacteria (LAB), propionic acid bacteria [35,39,40,41], *Bacillus* spp. [42,43], and yeast [44,45] has been described in multiple studies. However, the bio-detoxification of the emerging *Fusarium* mycotoxin ENB has not yet been studied extensively. In an earlier study, LAB and yeast were shown to reduce the content of different enniatins in culture medium in vitro [46], but to the best of our knowledge, only one study has been conducted on the ability of *Bacillus* spp. to degrade specifically ENB [47].

As different *Fusarium* species coexist on the crop fields, naturally, a wide range of *Fusarium* toxins are present in a single crop sample [48,49,50]. *F. graminearum* (DON producer) and *F. avenaceum* (ENB producer) are widely distributed in the fields, and therefore, enniatins, and in particular ENB and DON, often show co-occurrence in raw samples, food, and feed [6,17,51,52,53,54]. Both toxins have shown harmful impacts on gut health and immunological parameters in pigs [55]. Finding a suitable strategy to reduce the mycotoxin contamination in cereals is of particular importance for the consumer’s safety and for reducing the amount of food waste in the future. This is particularly important in the context of climate change, where the warmer temperatures are expected to increase the abundance of mycotoxin-producing fungi as well as to change the interaction between the different species, changing, therefore, the prevalence and the co-occurrence of mycotoxins found in the cereal’s fields in the future [56].

Therefore, the aim of this study was to evaluate strains of LAB and *Bacillus* spp., isolated from plants and with qualified presumption of safety (QSP) status according to the European Food Safety Authority (EFSA) [57], for their ability to reduce one of the most frequently detected emerging mycotoxins, ENB, and one of the most prevalent regulated mycotoxins, DON, in a cereal-based medium in vitro. To achieve this, a targeted HPLC-MS/MS approach was developed to quantify both ENB and DON in a liquid culture medium. A possible application of the ENB- and DON-reducing microorganisms found would be foreseen as a pre-treatment step for contaminated whole wheat kernels or wheat flours to enable their safe reintroduction into the food and feed value chain [30]. 

## 2. Materials and Methods

### 2.1. Standards and Chemicals

The mycotoxins ENB and DON were obtained from Merck (Sigma-Aldrich, E5411 and Supelco, CRM46911; Merck AG, Zug, Switzerland). All solvents and mobile phase modifiers were of LC-MS grade. Water and acetonitrile were supplied by Carl Roth AG (Arlesheim, Switzerland). Methanol was supplied by Honeywell Deutschland Holding GmbH (Offenbach, Germany). Formic acid, acetic acid, and ammonium formate were purchased from Sigma-Aldrich (Merck AG, Zug, Switzerland). MRS broth was obtained from VWR (Radnor, PA, USA) and BHI broth from Biolife Italiana (Monza, Italy). All sugars, sodium chloride, and yeast extract were supplied from Carl Roth AG (Arlesheim, Switzerland). Peptone was purchased from Biolife Italiana (Monza, Italy).

### 2.2. Microorganisms Used in the Study

In total, 238 LAB and *Bacillus* spp. strains with qualified presumption of safety (QPS) status were screened for ENB and DON reduction potential. The strains included isolates from the culture collection of the Food Biotechnology Research Group of ZHAW (Wädenswil, Switzerland) previously isolated from malt, spent grain, mycotoxin-contaminated grains, sourdough, convenient salads, kunu zaki, raw milk, yellow peas, and baker’s yeast [38,58,59]. The tested strains belonged to the following species: *Bacillus flexus* (1), *Bacillus licheniformis* (42), *Bacillus megaterium* (13), *Bacillus pumilus* (2), *Bacillus subtilis* (9), *Levilactobacillus brevis* (13), *Lacticaseibacillus casei* (1), *Lapidilactobacillus concavus* (3), *Loigolactobacillus coryniformis* (38), *Latilactobacillus curvatus* (12), *Limosilactobacillus fermentum* (1), *Lentilactobacillus kefiri* (1), *Lentilactobacillus parabuchneri* (4), *Lacticaseibacillus paracasei* (1), *Lactiplantibacillus plantarum* (7), *Limosilactobacillus reuteri* (1), *Lacticaseibacillus rhamnosus* (1), *Fructilactobacillus sanfranciscensis* (3), *Lactococcus lactis* (3), *Leuconostoc citreum* (19), *Leuconostoc lactis* (14), *Leuconostoc mesenteroides* (1), *Leuconostoc palmae* (2), *Leuconostoc pseudomesenteroides* (5), *Pediococcus acidilactici* (15), *Pediococcus pentosaceus* (22), *Weissella cibaria* (1), *Weissella confusa* (2), and an unidentified strain (1). 

### 2.3. Screening for Enniatin B and Deoxynivalenol Reduction

The screening for ENB and DON reduction was performed according to Mischler et al. [38]. Wheat flour hydrolysate medium (WFH) was prepared by mixing 200 g of wheat flour type 550 (Meyerhans Mühlen AG, Weinfelden, Switzerland) with 800 mL tap water and incubation at 30 °C and 90 rpm for 4 h. The suspension was stored in the fridge (4 °C) overnight (18 h), and the supernatant was used as wheat flour hydrolysate. WFH medium was made by the addition of 10 g yeast extract to 1 L of wheat flour hydrolysate. After sterilization (121 °C, 15 min), sugars were added (15 g glucose, 15 g maltose, 15 g sucrose, 15 g fructose), pH was set to 5.6, and the medium was sterile filtered.

Overnight cultures of the strains were prepared in MRS broth or BHI broth at 30 °C for LAB or *Bacillus* spp. strains, respectively. The overnight cultures were washed twice with diluent (1 g/L peptone, 8.5 g/L sodium chloride; 8000× *g*, 5 min) followed by resuspending them in diluent. One percent of these cultures were used for inoculation of 1.5 mL of WFH medium supplemented with either 300 ng/mL ENB (200 µg/mL in acetonitrile) or DON (200 μg/mL in ethyl acetate/methanol (95:5)). An uninoculated negative control was prepared by adding 1% of diluent to the WFH medium with the respective mycotoxin. All samples were incubated at 30 °C for 72 h. Prior to HPLC-MS/MS quantification of ENB and DON, the samples were shortly centrifuged (~30 s) and filtered (0.2 µm RC filters; Phenomenex, Torrance, CA, USA). The experiment was performed once for all 238 strains (n = 1). Positive strains (26 strains, >20% reduction in DON or >70% reduction in ENB) were confirmed in three additional runs of the experiment (number of independent replicates for each positive strain n = 4). 

### 2.4. HPLC-MS/MS Analysis of Enniatin B and Deoxynivalenol

Analyses were conducted on a system consisting of a Thermo Vanquish Horizon chromatographic system coupled with an Altis Triple Quadrupole (TQ) mass spectrometer equipped with an electrospray ionization source (ESI) (Thermo Fisher Scientific, Reinach, Switzerland). Chromatographic separation was performed using an Agilent Poroshell 120 EC-C18 (2.1 × 100 mm, 2.7 µm) column protected by a guard column (Agilent EC-18, 2.1 × 5 mm, 2.7 µm) (Agilent, Technologies, Basel, Switzerland). The two mobile phases consisted of water + 2% methanol + 5 mM ammonium formate + 0.1% formic acid + 0.1% acetic acid (mobile phase A) and methanol + 2% water + 5 mM ammonium formate + 0.1% formic acid + 0.1% acetic acid (mobile phase B). The flow rate was set to 0.3 mL/min, the column temperature at 40 °C with forced air option on, and precolumn heater at 40 °C. The gradient was set as follows: 0–0.5 min, 5% B; 6–14 min, 98% B; and 14.1–18 min, 5% B. Injection volume was 5 µL.

The MS analyses were performed using the Thermo Altis triple Quadrupole instrument in positive ionization mode (ESI+) and in selected reaction monitoring (SRM) mode, alternating 3 transition reactions with the following settings: nitrogen served as nebulizing gas and argon as collision gas. The spray voltage was set to 3000 V, sheath gas was set at 40 (Arb), aux gas at 18 (Arb), sweep gas at 20 (Arb), and ion transfer tube temperature at 350 °C. Vaporizer temperature was set at 300 °C and CID gas at 0.5 mTorr.

MS tuning and optimization of the MS/MS parameters for ENB and DON analysis were performed by means of direct infusion with a syringe of a separate standard solution of DON (2 µg/mL) and ENB (1 µg/mL) into the TQ using a syringe pup at a flow rate of 5 µL/min.

Optimized ESI-MS and ESI-MS/MS parameters and monitored transition reactions for ENB and DON are summarized in Table 1. For accurate quantification, a matrix-matched calibration was carried out by spiking the ENB and DON at 7 different concentrations in the blank medium WFH. Quantification parameters for each mycotoxin are given in Table 1.

Intraday relative standard deviations (RSD) were calculated by injecting three different control vials with known amounts of ENB and DON in WFH medium at the beginning of the day and again at the end of the day. Interday relative standard deviations were calculated by injecting the same vials on the next day (vials were kept at −20 °C overnight). Intraday RSD of the method was 4.3% for DON and 7.6% for ENB. Interday RSD of the method was 7.2% for DON and 3.9% for ENB. 

### 2.5. Statistics

Statistical analysis was conducted using the XLSTAT statistical and data analysis solution for Excel (Premium Edition 2023.1.6, Lumivero, Denver, CO, USA). Inoculated samples were compared to their respective control using an ANOVA with Dunnett pairwise comparison (alpha = 5%; n = 4).

## 3. Results

### 3.1. Screening for Bio-Detoxification Potential of Lactic Acid Bacteria and Bacillus spp. for Enniatin B and Deoxynivalenol

In a first step, a total number of 238 LAB and *Bacillus* spp. strains were screened for ENB or DON reduction potential. Table 2 shows the reduction potential of ENB by the tested strains, classified by species, indicating the number of strains able to reduce ENB by more than 90%, between 70 and 90%, and less than 20% after 72 h of incubation at 30 °C (see Table S1 in the Supplementary Materials for the detailed activity of each of the 238 strains tested).

Of the 238 strains tested for ENB reduction, 9 strains of *B. licheniformis* showed a reduction of more than 90%, and 2 strains of each *B. licheniformis* and *B. pumilus* revealed a reduction in ENB between 70 and 90%. All other tested strains (225) showed a reduction in ENB below 20%.

Table 3 shows the DON reduction potential of the tested strains by species, indicating the number of strains able to reduce DON by 30 to 40%, 20 to 30%, 10 to 20%, and less than 10% after 72 h of incubation at 30 °C (see Table S1 in the Supplementary Materials for the detailed activity of each of the 238 strains tested).

Of the 238 strains tested, one *B. licheniformis* strain showed a 32% reduction in DON after 72 h incubation at 30 °C. Additionally, 18 further *B. licheniformis* strains showed a reduction of 20 to 30%. A group of 25 strains of different species revealed a capacity to reduce DON by 10 to 20%: 17 *B. licheniformis* strains, 1 *B. subtilis* strain, 4 *L. coryniformis* strains, 2 *Lc. lactis* strains, and 1 *Ln. lactis* strain. All other strains showed a reduction below 10%.

Overall, 26 best-performing strains, 2 *Bacillus pumilus* and 24 *Bacillus licheniformis*, were selected based on their ability to reduce DON (>20% reduction) and/or ENB (>70% reduction) for further evaluation in a repeated screening in order to confirm their activity in three independent cultivations.

### 3.2. Detailed Analysis of the Most Promising Bio-Detoxifying Strains for Enniatin B and Deoxynivalenol Reduction

**Figure 1:**
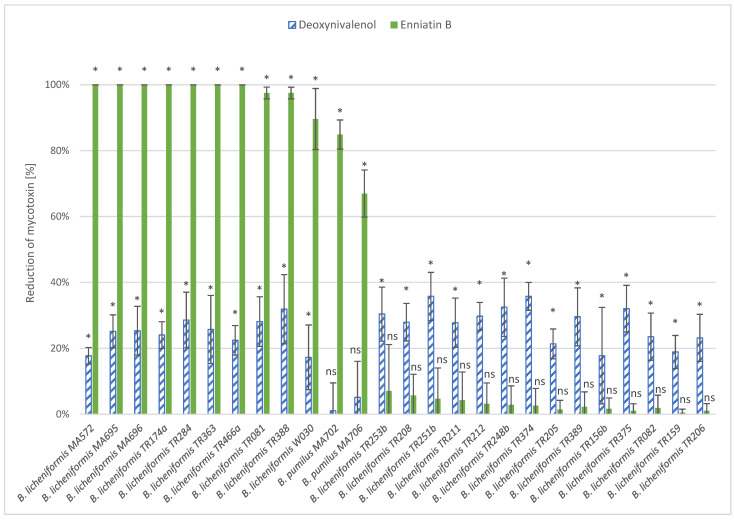
The reduction in DON and ENB [%], respectively, after the incubation of the 26 selected strains in WFH medium supplemented with 300 ng/mL of ENB (green, filled) or DON (blue, stripes) compared to an uninoculated control (n = 4). Statistics: ANOVA with Dunnett pairwise comparison with control, * *p* < 0.05, ns not significant.

The 26 best-performing strains determined in the screening experiments (Section 3.1) were cultivated in triplicates, as in the screening, in order to confirm their bio-detoxification potential. Figure 1 shows the reduction (%) of the mycotoxins ENB and DON in the cultivation medium after incubation with the 26 selected strains (average values and standard deviations are available in Table S2 of the Supplementary Materials).

The incubation of *B. licheniformis* strains MA572, MA695, MA696, TR174a, TR284, TR363, and TR466a with ENB for 72 h led to a reduction to values below that of the limit of detection of 0.05 ng/mL (* significant difference with control, ANOVA α = 5%, Dunnett pairwise comparison). Moreover, a significant reduction in ENB was confirmed for the *B. licheniformis* strains W030, TR081, and TR388 (reduction of 89.6%, 97.5%, and 97.5%, respectively) and the *B. pumilus* strain MA702 (reduction of 84.9%). MA706 showed a significant reduction of 67% of the initial ENB concentration.

The two *B. pumilus* strains MA702 and MA706 showed no significant reduction in DON. On the contrary, all the other tested strains showed a significant reduction in DON with an average of 26.4%. The most promising DON-reducing strains were *B. licheniformis* TR248b, TR251b, TR253b, TR374, TR375, and TR388, which showed 30 to 40% DON reduction, whereas *B. licheniformis* TR251b and TR374 were the most effective strains with a 35.7% reduction in DON.

## 4. Discussion

In this study, a successful microbial reduction of the most prevalent emerging mycotoxin ENB was revealed. This is, to the best of our knowledge, one of the first studies screening for ENB reduction by microbial fermentation, along with Meca et al. [47] and Roig et al. [46]. In total, 238 strains of lactic acid bacteria (LAB) and *Bacillus* spp. were incubated at 30 °C for 72 h in a cereal-based broth (wheat flour hydrolysate) containing 300 ng/mL of ENB. Among all strains tested, *B. licheniformis* MA572, MA695, MA696, TR174a, TR284, TR363, and TR466a showed a 100% reduction in ENB (reduction in ENB below the limit of detection of 0.05 ng/mL); the fermentation with *B. licheniformis* W030, TR081, and TR388 led to an 89%, 97.5%, and 97.5% reduction in ENB, respectively; and an 85% reduction was reached with *B. pumilus* MA702. To the best of our knowledge, this is the first time that the detoxification potential of *B. licheniformis* strains has been reported. However, all the LAB strains tested proved to be ineffective in reducing ENB. 

Meca et al. [47] and Roig et al. [46] revealed similar reduction levels as determined in this study. Roig et al. investigated *Bifidobacterium* spp. strains, LAB strains, and yeast strains of *S. cerevisiae*, demonstrating that some yeast could reduce over 90% of the ENB. Further, strains of *Streptococcus thermophilus*, *L. rhamnosus*, and *Bifidobacterium adolescentis* showed a reduction in ENB of over 70% [46]. Unlike the data presented by Roig et al., the *L. rhamnosus* strain tested in the current study was shown to be ineffective in reducing ENB. On the other hand, Meca et al. [47] demonstrated a reduction in ENB of >90% by strains of *B. subtilis*. In contrast, our investigation revealed a limited ENB reduction of less than 20% for the nine tested strains of *B. subtilis.*

Different experimental conditions, namely culture media, incubation temperature, and starting CFU/mL, were applied in the current study in comparison with the studies of Meca and Roig. In this study, a cereal-based medium (wheat flour hydrolysate) combined with aerobic incubation at 30 °C were selected, aiming at simulating the potential real-life conditions of this technique to detoxify wheat flour or wheat grains in the future. The experimental conditions chosen by Meca et al. were the use of a rich nutrient broth, i.e., tryptic soy broth, as well as higher incubation temperatures (37 °C vs. 30 °C) and a higher starting inoculum (10^8^ CFU/mL vs. 10^5^ CFU/mL), whereas the microorganisms tested in the current study had poorer conditions to grow under, and therefore, their detoxifying abilities may have been influenced, which could explain why the *B. subtilis* strains screened were less efficient compared to those described by Meca et al. [47]. 

As for ENB reduction, the 238 strains were screened for DON reduction potential by applying the same approach and using the same toxin levels. Twenty-four *B. licheniformis* strains could reduce the DON concentration by at least 20%. *B. licheniformis* strains TR248b, TR251b, TR253b, TR374, TR375, and TR388 had the biggest effect on DON levels with a 30 to 40% reduction. *B. licheniformis* TR251b and TR374 were the most effective with a 35.7% reduction in DON. None of the tested LAB strains were efficient in reducing DON. 

Other studies have demonstrated the reduction of the mycotoxin DON by *Bacillus* spp. strains, such as Cheng et al. [60], who revealed that a strain of *B. subtilis* could reduce DON by 98% and a strain of *B. licheniformis* by 72%. Additionally, Jia et al. [42] found a *B. subtilis* strain with an 80% reduction potential of DON, and Wang et al. [61] determined an 80% DON reduction by a *B. licheniformis* strain after 48 h of fermentation.

On the other hand, in the group of LAB, a study from Chlebicz and Śliżewska [44] uncovered *L. paracasei*, *L. pentosus*, *L. plantarum*, *L. reuteri*, and *L. rhamnosus* strains showing a 30–40% reduction in DON after 24 h of fermentation. In addition, Franco et al. [39] found a *L. paracasei* strain with a reduction potential of 30% and *L. plantarum* strains that could reduce DON by 50%. Further, Zhai et al. [62], discovered a 40% DON reduction by the cell wall of *L. paracasei*. In all mentioned studies, the experimental conditions used are different compared to the current study, with MRS broth as a cultivation medium and a washing of the cells with phosphate-buffered saline (PBS) prior to contact with the mycotoxin. In addition, the incubation temperature was set at 37 °C, whereas in the current study, a lower temperature of 30 °C was applied. 

A strong correlation between ENB and DON contamination has recently been found in cereal-based products [6]. Therefore, microbial strains that show a natural reduction of both mycotoxins could be a first step to new and future-orientated bio-detoxification strategies. In this study, we highlight an effective approach to reduce both mycotoxins with the use of *B. licheniformis* TR284 (DON reduction of 28.6%, ENB reduction of 100%) or *B. licheniformis* TR388 (DON reduction of 31.9%, ENB reduction of 97.5%). Both strains were isolated from spent grain and are part of the culture collection of the Food Biotechnology Research Group of ZHAW (Wädenswil, Switzerland). Spent grain is known to be susceptible to mycotoxin contamination [63,64]. Therefore, the source of isolation, i.e., a mycotoxin-loaded substrate, targeting microorganisms with bio-detoxification potential can be an advantage in the approach to reduce mycotoxins in cereal products by fermentation since the applied strains are natural inhabitants of the substrate, and the assumption can be made that their metabolism is adapted to the components present, including mycotoxins. Furthermore, *B. licheniformis* belongs to the species with QPS status and are therefore safe for use in food applications as long as the microorganisms do not have any antimicrobial resistances and do not show any toxigenic activity [65]. This would be a valuable advantage in real-case application to bio-detoxify wheat flours or wheat grains using selected strains of *B. licheniformis*. A potential strategy to reduce mycotoxin levels in wheat kernels was previously explored in a study by André et al. [30]. In this study, wheat kernels were subjected to cold needle perforation and subsequently incubated with mycotoxin-reducing bacilli. However, André et al. observed no significant effect of the bacilli on the kernels, indicating that further research is necessary to bridge the gap between in vitro trials and in situ applications.

So far, the mechanism of the bio-detoxification [46] of the strains presented in the current study is not known. In the case of a later application of these strains in bio-detoxification strategies of food and feedstuff, this knowledge would be mandatory, i.e., if the reduction observed is caused by a binding to the bacterial cell wall or by a degradation of the toxin due to the activity of the bacterial enzymes. A binding mechanism would be problematic since either the mycotoxin could be released from the bacterial cell wall after ingestion or, in the case of degradation, the resulting compounds must not be toxic [36,37]. Therefore, ongoing work is being conducted in order to understand the mechanism of action of the microorganisms in the view of developing concurrent bio-detoxification strategies for ENB and DON in cereal-based products. In addition, future studies should consider the application of mycotoxin-reducing strains to contaminated whole wheat grains or flour for bio-detoxification. A major limitation of this decontamination strategy could be an insufficient growth of the microorganisms on the wheat kernels as well as the reduced activity of the positive strains due to low water availability [30]. Therefore, optimal parameters for the growth of the microorganisms have to be further investigated. Overall, a biological treatment with microorganisms would have high specificity and efficiency and would result in environmentally friendly products compared to physical treatments [29]. The bio-detoxification of mycotoxin-contaminated grains would be important for the reduction of food losses, the improvement of food safety, and the health aspects of the final products [33].

## 5. Conclusions

This study showed the successful reduction of ENB and DON in vitro by *Bacillus* spp. strains. Out of 238 strains, 2 *B. pumilus* and 24 *B. licheniformis* strains showed high mycotoxin reduction potential. In particular, *B. licheniformis* TR284 with 100% ENB and 28.6% DON reduction each and *B. licheniformis* TR388 with a 97.5% and 31.9% reduction in ENB and DON, respectively, could be used in a bio-detoxification strategy to minimize mycotoxin levels in whole wheat kernels or flour. The application of these promising strains to food raw materials would be an important next step in future research. The bio-detoxification approach would be valuable for the reintroduction of safer food products into the food and feed value chain and therefore reducing food waste.

## Figures and Tables

**Table 1:** ESI-MS and ESI-MS/MS parameters and monitored transition reactions for the analysis of ENB and DON.

Compounds	Retention Time (min)	Precursor Ion (*m*/*z*)	Adduct Ion	RF Lens (V)	Product Ions (*m*/*z*)	Collision Energy (V)	Calibration: Linear Range (ng/mL)	Calibration Equation and R^2^ Factor	LOD (ng/mL)	LOQ (ng/mL)
ENB	7.18	640.23	[M + H]^+^	86	195.967 (quan)	28.5	1–500	y = 7132.9x − 329,488 R^2^ = 0.9986	0.05	1
					213.967 (qual)	28.3				
					527.16 (qual)	24.8				
DON	3.27	296.967	[M + H]^+^	38	248.967 (quan)	15.6	1–1000	y = 225.07x + 209.65 R^2^ = 0.9994	0.1	1
					202.967 (qual)	20.7				
					230.883 (qual)	18.1				

**Table 2:** Total number of screened strains of LAB and *Bacillus* spp. with reduction in ENB (300 ng/mL) of >90%, 70–90%, and <20% after 72 h incubation at 30 °C (n = 1).

ENB Reduction				
Species	Total Screened	>90%	70–90%	<20%
*B. flexus*	1	0	0	1
*B. licheniformis*	42	9	2	31
*B. megaterium*	13	0	0	13
*B. pumilus*	2	0	2	0
*B. subtilis*	9	0	0	9
*L. brevis*	13	0	0	13
*L. casei*	1	0	0	1
*L. concavus*	3	0	0	3
*L. coryniformis*	38	0	0	38
*L. curvatus*	12	0	0	12
*L. fermentum*	1	0	0	1
*L. kefiri*	1	0	0	1
*L. parabuchneri*	4	0	0	4
*L. paracasei*	1	0	0	1
*L. plantarum*	7	0	0	7
*L. reuteri*	1	0	0	1
*L. rhamnosus*	1	0	0	1
*F. sanfranciscensis*	3	0	0	3
*Lc. lactis*	3	0	0	3
*Ln. citreum*	19	0	0	19
*Ln. lactis*	14	0	0	14
*Ln. mesenteroides*	1	0	0	1
*Ln. palmae*	2	0	0	2
*Ln. pseudomesenteroides*	5	0	0	5
*P. acidilactici*	15	0	0	15
*P. pentosaceus*	22	0	0	22
*W. cibaria*	1	0	0	1
*W. confusa*	2	0	0	2
no identification	1	0	0	1
Total	238	9	4	225

**Table 3:** Total number of screened strains of LAB and *Bacillus* spp. with reduction in DON (300 ng/mL) of 30–40%, 20–30%, 10–20%, and <10% after 72 h incubation at 30 °C (n = 1).

DON Reduction					
Species	Total Screened	30–40%	20–30%	10–20%	<10%
*B. flexus*	1	0	0	0	1
*B. licheniformis*	42	1	18	17	6
*B. megaterium*	13	0	0	0	13
*B. pumilus*	2	0	0	0	2
*B. subtilis*	9	0	0	1	8
*L. brevis*	13	0	0	0	13
*L. casei*	1	0	0	0	1
*L. concavus*	3	0	0	0	3
*L. coryniformis*	38	0	0	4	34
*L. curvatus*	12	0	0	0	12
*L. fermentum*	1	0	0	0	1
*L. kefiri*	1	0	0	0	1
*L. parabuchneri*	4	0	0	0	4
*L. paracasei*	1	0	0	0	1
*L. plantarum*	7	0	0	0	7
*L. reuteri*	1	0	0	0	1
*L. rhamnosus*	1	0	0	0	1
*F. sanfranciscensis*	3	0	0	0	3
*Lc. lactis*	3	0	0	2	1
*Ln. citreum*	19	0	0	0	19
*Ln. lactis*	14	0	0	1	13
*Ln. mesenteroides*	1	0	0	0	1
*Ln. palmae*	2	0	0	0	2
*Ln. pseudomesenteroides*	5	0	0	0	5
*P. acidilactici*	15	0	0	0	15
*P. pentosaceus*	22	0	0	0	22
*W. cibaria*	1	0	0	0	1
*W. confusa*	2	0	0	0	2
no identification	1	0	0	0	1
Total	238	1	18	25	194

## Data Availability

The original contributions presented in the study are included in the article/Supplementary Material; further inquiries can be directed to the corresponding author.

## Supplementary Materials

The following supporting information can be downloaded at https://www.mdpi.com/article/10.3390/microorganisms12091892/s1, Table S1: Screening of 238 strains for reduction of DON and ENB after 72 h of incubation at 30 °C in cereal based culture medium (n = 1), in comparison with control.; Table S2: Reduction of DON and ENB [%], respectively, after incubation of the 26 selected strains in WFH medium supplemented with 300 ng/mL of DON or ENB compared to a non-inoculated control (n = 4).
