# Supplementary material for: Potential for the Bio-Detoxification of the Mycotoxins Enniatin B and Deoxynivalenol by Lactic Acid Bacteria and Bacillus spp
Source: Microorganisms. 2024 Sep 13;12(9):1892. doi: 10.3390/microorganisms12091892 (PMC11434589; doi:10.3390/microorganisms12091892)
Supplement: Supplementary file 1 [file microorganisms-12-01892-s001.zip › microorganisms-3192250-supplementary.pdf]

Supplementary Material:

Potential for bio-detoxification of the mycotoxins enniatin B and deoxynivalenol by lactic acid bacteria and *Bacillus* spp.

Sandra Mischler, Amandine André, Irene Chetschik and Susanne Miescher Schwenninger

Institute of Food and Beverage Innovation, ZHAW Zurich University of Applied Sciences, 8820 Wädenswil, Switzerland

Table S1: Screening of 238 strains for reduction of DON and ENB after 72 hours of incubation at 30°C in cereal based culture medium (n=1), in comparison with control.

Strains	% reduction DON	% reduction ENB
Weissella cibaria Myk001	1.4%	0.0%
Lentilactobacillus kefiri Myk025	0.0%	0.0%
Pediococcus pentosaceus Myk040	0.0%	0.0%
Latilactobacillus curvatus Myk043	0.0%	0.0%
Latilactobacillus curvatus Myk049	0.0%	0.0%
Pediococcus pentosaceus Myk055	0.0%	0.0%
Pediococcus pentosaceus Myk058	0.0%	0.0%
Pediococcus acidilactici Myk066	0.0%	0.0%
Pediococcus pentosaceus Myk079	0.0%	0.0%
Loigolactobacillus coryniformis Myk084	0.0%	0.0%
Pediococcus pentosaceus Myk089	0.0%	0.0%
Loigolactobacillus coryniformis Myk099	0.0%	0.0%
Pediococcus acidilactici Myk130	0.0%	0.0%
Loigolactobacillus coryniformis Myk140	0.0%	0.0%
Pediococcus pentosaceus Myk149	0.0%	0.0%
Lactococcus lactis Myk163	0.0%	0.0%
Pediococcus pentosaceus Myk165	0.0%	0.0%
Pediococcus pentosaceus Myk167	0.0%	0.0%
Loigolactobacillus coryniformis Myk004	0.0%	0.0%
Loigolactobacillus coryniformis Myk017	0.0%	0.0%
Loigolactobacillus coryniformis Myk018	0.0%	0.0%
Loigolactobacillus coryniformis Myk023	0.0%	0.0%
Lapidilactobacillus concavus Myk026	0.0%	0.0%
Loigolactobacillus coryniformis Myk030	0.0%	0.0%
Lapidilactobacillus concavus Myk033	0.0%	0.0%
Loigolactobacillus coryniformis Myk042	0.0%	0.0%

Loigolactobacillus coryniformis Myk044	0.0%	0.0%
Pediococcus pentosaceus Myk047	0.0%	0.0%
Loigolactobacillus coryniformis Myk048	0.0%	2.4%
Loigolactobacillus coryniformis Myk052	0.0%	7.7%
Loigolactobacillus coryniformis Myk057	0.8%	7.2%
Pediococcus pentosaceus Myk062	0.8%	5.9%
Loigolactobacillus coryniformis Myk063	0.0%	0.0%
Loigolactobacillus coryniformis Myk064	0.0%	0.5%
Loigolactobacillus coryniformis Myk071	1.9%	5.3%
Loigolactobacillus coryniformis Myk073	0.0%	0.6%
Loigolactobacillus coryniformis Myk075	0.0%	0.0%
Loigolactobacillus coryniformis Myk080	0.0%	0.0%
Pediococcus pentosaceus Myk082	0.0%	0.0%
Loigolactobacillus coryniformis Myk088	0.0%	0.0%
Lapidilactobacillus concavus Myk092	0.0%	0.0%
Loigolactobacillus coryniformis Myk095	0.3%	0.0%
Loigolactobacillus coryniformis Myk097	0.0%	0.0%
Loigolactobacillus coryniformis Myk100	0.0%	0.0%
Pediococcus pentosaceus Myk113	0.0%	0.0%
Loigolactobacillus coryniformis Myk114	0.0%	0.0%
Pediococcus pentosaceus Myk116	0.0%	0.0%
Pediococcus pentosaceus Myk123	0.0%	0.0%
Pediococcus pentosaceus Myk152	0.0%	0.0%
Pediococcus pentosaceus Myk156	0.0%	0.0%
Pediococcus pentosaceus Myk169	5.6%	1.6%
Pediococcus pentosaceus Myk171	6.1%	0.0%
Pediococcus pentosaceus Myk172	8.6%	1.5%
Pediococcus pentosaceus Myk189	8.4%	1.9%
Levilactobacillus brevis JR1	0.0%	0.0%
Levilactobacillus brevis JR6	0.0%	0.0%
Levilactobacillus brevis JR11	0.0%	0.0%
Levilactobacillus brevis JR13	0.0%	0.0%
Levilactobacillus brevis JR70	0.0%	0.0%
Levilactobacillus brevis JR98	0.0%	0.0%
Levilactobacillus brevis JR114	0.0%	0.0%
Lentilactobacillus parabuchneri JR149	0.0%	0.0%
Lentilactobacillus parabuchneri JR158	0.0%	0.0%
Lentilactobacillus parabuchneri JR182	0.0%	0.0%
Levilactobacillus brevis JR187	0.0%	0.0%
Lentilactobacillus parabuchneri JR194	0.0%	0.0%
Levilactobacillus brevis JR196	0.0%	0.0%
Leuconostoc citreum MA079	0.7%	2.5%
Leuconostoc citreum MA083	3.0%	0.0%
Leuconostoc citreum MA105	0.7%	0.0%
Leuconostoc citreum MA106	0.0%	0.0%

Leuconostoc citreum MA108	0.0%	2.5%
Leuconostoc citreum MA110	0.8%	5.0%
Leuconostoc citreum MA113	8.1%	0.8%
Leuconostoc citreum MA189	1.3%	6.4%
Leuconostoc citreum MA268	5.1%	1.6%
Levilactobacillus brevis MA278b	0.0%	0.0%
Limosilactobacillus fermentum MSB-U_pi-I11	0.0%	0.0%
Bacillus megaterium Myk106	0.0%	0.0%
Bacillus megaterium Myk108	3.5%	0.0%
Bacillus megaterium Myk110	0.0%	0.0%
Bacillus megaterium Myk118	0.4%	0.0%
Bacillus megaterium Myk121	0.0%	0.0%
Bacillus megaterium Myk132	5.1%	0.0%
Bacillus megaterium Myk133	0.0%	0.0%
Bacillus megaterium Myk135	0.0%	0.0%
Bacillus megaterium Myk145	1.5%	0.0%
Bacillus megaterium Myk147	0.0%	0.0%
Bacillus megaterium Myk148	0.0%	0.0%
Bacillus licheniformis TR078	17.9%	0.0%
Bacillus licheniformis TR081	29.6%	100.0%
Bacillus licheniformis TR082	28.7%	0.0%
Bacillus licheniformis TR086	19.0%	0.0%
Bacillus licheniformis TR103	0.0%	0.0%
Bacillus licheniformis TR156b	22.5%	0.0%
Bacillus licheniformis TR159	23.4%	0.0%
Bacillus licheniformis TR174a	25.9%	100.0%
Bacillus licheniformis TR205	22.1%	0.0%
Bacillus licheniformis TR206	26.2%	0.0%
Bacillus licheniformis TR208	25.4%	0.0%
Bacillus licheniformis TR211	23.0%	0.0%
Bacillus licheniformis TR212	27.0%	0.0%
Bacillus licheniformis TR253b	20.9%	0.0%
Bacillus flexus TR372	0.0%	3.4%
Bacillus licheniformis TR253a	18.7%	11.0%
Bacillus licheniformis TR252	17.3%	4.6%
Bacillus licheniformis TR363	13.5%	100.0%
Bacillus megaterium TR362	0.5%	7.2%
Bacillus megaterium TR354	5.2%	9.6%
Bacillus subtilis TR351	11.4%	0.0%
Bacillus licheniformis TR284	17.6%	100.0%
Bacillus licheniformis TR254	18.6%	18.7%
Bacillus licheniformis TR251b	27.1%	0.0%
Bacillus licheniformis TR251a	17.2%	10.1%
Bacillus licheniformis TR250b	17.4%	6.0%
Bacillus licheniformis TR250a	18.6%	6.8%

Bacillus licheniformis TR248b	21.1%	0.0%
Bacillus licheniformis TR248a	15.7%	4.5%
Bacillus licheniformis TR466a	20.4%	100.0%
Bacillus pumilus MA706	0.0%	73.2%
Bacillus subtilis MA705	0.5%	0.0%
Bacillus pumilus MA702	0.0%	79.4%
Bacillus licheniformis MA697	6.4%	0.0%
Bacillus licheniformis MA696	23.7%	100.0%
Bacillus licheniformis MA695	22.1%	100.0%
Bacillus licheniformis MA572	16.2%	100.0%
Bacillus licheniformis TR389	20.1%	0.0%
Bacillus licheniformis TR388	19.0%	100.0%
Bacillus licheniformis TR376	8.0%	3.6%
Bacillus licheniformis TR375	23.9%	0.0%
Bacillus licheniformis TR374	31.7%	0.0%
Bacillus licheniformis TR373	14.0%	0.0%
Bacillus licheniformis MA693b	13.7%	0.0%
Lactiplantibacillus plantarum MA451	0.0%	0.0%
Leuconostoc citreum MA437	0.0%	0.0%
Leuconostoc citreum MA436	0.0%	1.3%
Leuconostoc citreum MA432	1.2%	9.0%
Lactiplantibacillus plantarum MA422	0.0%	0.0%
Leuconostoc citreum DCM83	0.0%	5.2%
Leuconostoc citreum DCM74	2.2%	0.4%
Leuconostoc citreum DCM65	3.9%	3.5%
Leuconostoc citreum DCM63	0.2%	6.6%
Weissella confusa DCM53	0.0%	0.0%
Leuconostoc citreum MA419	0.0%	9.9%
Leuconostoc pseudomesenteroides MA407	0.0%	0.0%
Leuconostoc pseudomesenteroides MA072	0.0%	0.0%
Leuconostoc palmae DCM49	0.0%	0.0%
Leuconostoc mesenteroides DCM27	0.0%	0.0%
Leuconostoc citreum DCM3	0.0%	1.8%
Leuconostoc palmae DCM85	0.3%	1.0%
not identified JR33	0.0%	0.0%
Leuconostoc lactis TR249b	0.0%	0.0%
Leuconostoc lactis TR249a	11.5%	0.0%
Leuconostoc lactis TR181	0.2%	1.7%
Leuconostoc lactis TR180	0.0%	6.4%
Leuconostoc lactis TR145a	0.7%	1.3%
Leuconostoc lactis TR143a	0.0%	0.0%
Leuconostoc lactis TR142b	3.1%	2.3%
Leuconostoc lactis TR141b	0.0%	2.5%
Leuconostoc lactis TR071	0.1%	2.4%
Leuconostoc lactis TR179	0.0%	6.5%

Leuconostoc lactis TR158	0.0%	1.0%
Leuconostoc lactis TR146a	0.0%	0.0%
Leuconostoc lactis TR145b	0.0%	0.0%
Leuconostoc pseudomesenteroides MA457	0.0%	0.0%
Leuconostoc lactis TR067	0.0%	0.0%
Leuconostoc citreum MA663a	0.0%	2.1%
Weissella confusa MA463	0.0%	0.0%
Pediococcus pentosaceus Myk014	0.0%	2.7%
Pediococcus pentosaceus Myk016	0.0%	4.3%
Pediococcus acidilactici Myk019	0.0%	0.0%
Pediococcus acidilactici Myk022	0.0%	0.0%
Pediococcus acidilactici Myk029	0.0%	0.0%
Pediococcus acidilactici Myk032	0.0%	0.0%
Pediococcus acidilactici Myk035	0.0%	0.0%
Pediococcus acidilactici Myk036	0.0%	0.0%
Latilactobacillus curvatus Myk051	0.0%	2.0%
Loigolactobacillus coryniformis Myk054	0.0%	2.1%
Latilactobacillus curvatus Myk059	0.0%	14.8%
Latilactobacillus curvatus Myk061	0.0%	8.8%
Pediococcus acidilactici Myk067	0.0%	7.2%
Latilactobacillus curvatus Myk068	0.0%	5.0%
Latilactobacillus curvatus Myk069	0.0%	8.7%
Latilactobacillus curvatus Myk074	0.0%	0.0%
Pediococcus acidilactici Myk076	0.0%	8.1%
Pediococcus acidilactici Myk081	0.0%	17.4%
Pediococcus acidilactici Myk170	0.0%	5.2%
Latilactobacillus curvatus Myk083	0.0%	8.8%
Loigolactobacillus coryniformis Myk087	0.6%	10.2%
Latilactobacillus curvatus Myk090	0.0%	9.3%
Latilactobacillus curvatus Myk093	0.0%	12.1%
Latilactobacillus curvatus Myk094	0.0%	4.6%
Pediococcus acidilactici Myk124	0.0%	14.3%
Pediococcus acidilactici Myk129	0.0%	2.4%
Pediococcus acidilactici Myk168	0.0%	6.2%
Fructilactobacillus sanfranciscensis JR152a	0.0%	5.4%
Fructilactobacillus sanfranciscensis JR152b	0.0%	6.9%
Fructilactobacillus sanfranciscensis JR223a	0.0%	1.2%
Bacillus licheniformis TR278	7.4%	11.9%
Bacillus licheniformis TR387	13.2%	0.0%
Bacillus subtilis IB003	0.0%	3.6%
Bacillus licheniformis TR174a	0.0%	0.0%
Bacillus licheniformis W030	16.6%	77.2%
Bacillus licheniformis C115	5.0%	0.0%
Bacillus subtilis R072	5.6%	0.0%
Bacillus subtilis CS089	0.7%	0.0%

Bacillus subtilis CS101	0.0%	0.0%
Bacillus subtilis CS195	4.4%	0.0%
Bacillus subtilis CS690	5.5%	0.0%
Bacillus subtilis CS764	0.0%	0.0%
Lacticaseibacillus casei RMH002	0.0%	8.9%
Lacticaseibacillus rhamnosus RMH005	0.0%	0.0%
Lacticaseibacillus paracasei RMH007	0.0%	6.3%
Lactiplantibacillus plantarum SKMIM4	0.0%	4.8%
Lactiplantibacillus plantarum KMJIC6	0.0%	0.0%
Lactiplantibacillus plantarum YKDIA2	0.0%	0.0%
Lactiplantibacillus plantarum MKMIJ2	0.0%	6.5%
Lactiplantibacillus plantarum CR020	0.0%	3.5%
Limosilactobacillus reuteri CR047	0.0%	2.2%
Levilactobacillus brevis Hfe015	0.0%	4.2%
Levilactobacillus brevis JNT134	0.0%	0.0%
Loigolactobacillus coryniformis Myk011b	5.7%	0.0%
Lactococcus lactis Myk111a	11.4%	0.0%
Lactococcus lactis Myk111b	11.0%	0.0%
Loigolactobacillus coryniformis Myk115a	16.1%	0.0%
Loigolactobacillus coryniformis Myk115b	15.5%	0.0%
Loigolactobacillus coryniformis Myk128a	7.2%	0.0%
Loigolactobacillus coryniformis Myk128b	6.0%	0.0%
Loigolactobacillus coryniformis Myk141a	8.3%	0.0%
Levilactobacillus brevis JNT171	7.1%	0.0%
Loigolactobacillus coryniformis Myk011a	8.6%	0.0%
Loigolactobacillus coryniformis Myk151b	10.1%	0.0%
Leuconostoc pseudomesenteroides Myk153a	7.1%	0.0%
Leuconostoc pseudomesenteroides Myk153b	5.4%	0.0%
Loigolactobacillus coryniformis Myk184a	6.3%	0.0%
Loigolactobacillus coryniformis Myk184b	5.8%	0.0%
Loigolactobacillus coryniformis Myk141b	8.3%	0.0%
Loigolactobacillus coryniformis Myk151a	10.0%	0.0%

Table S2: Reduction of DON and ENB [%], respectively, after incubation of the 26 selected strains in WFH medium supplemented with 300 ng/ml of DON or ENB compared to a non-inoculated control (n=4).

	[%] mycotoxin reduction			
Strain	DON	DON standard deviation	ENB	ENB standard deviation
B. licheniformis MA572	17.74%	2.51%	100.00%	0.00%
B. licheniformis MA695	25.16%	4.97%	100.00%	0.00%
B. licheniformis MA696	25.35%	7.36%	100.00%	0.00%
B. licheniformis TR174a	24.06%	4.04%	100.00%	0.00%
B. licheniformis TR284	28.60%	8.43%	100.00%	0.00%
B. licheniformis TR363	25.72%	10.32%	100.00%	0.00%
B. licheniformis TR466a	22.45%	4.49%	100.00%	0.00%
B. licheniformis TR081	28.13%	7.51%	97.53%	1.79%
B. licheniformis TR388	31.87%	10.48%	97.52%	1.77%
B. licheniformis W030	17.28%	9.83%	89.61%	9.26%
B. pumilus MA702	1.05%	8.46%	84.90%	4.43%
B. pumilus MA706	5.11%	10.96%	66.99%	7.15%
B. licheniformis TR253b	30.37%	8.18%	7.10%	14.07%
B. licheniformis TR208	27.92%	5.67%	5.72%	6.41%
B. licheniformis TR251b	35.77%	7.27%	4.68%	9.36%
B. licheniformis TR211	27.77%	7.45%	4.27%	8.55%
B. licheniformis TR212	29.71%	4.19%	3.17%	6.34%
B. licheniformis TR248b	32.47%	8.82%	2.87%	5.75%
B. licheniformis TR374	35.74%	4.22%	2.61%	5.22%
B. licheniformis TR205	21.36%	4.54%	1.41%	2.81%
B. licheniformis TR389	29.57%	8.76%	2.27%	4.53%
B. licheniformis TR156b	17.76%	14.62%	1.65%	3.30%
B. licheniformis TR375	32.03%	7.05%	1.06%	2.13%
B. licheniformis TR082	23.52%	7.16%	1.93%	3.86%
B. licheniformis TR159	18.91%	5.02%	0.51%	1.02%
B. licheniformis TR206	23.16%	7.14%	1.08%	2.16%
